# Only fourteen 3′-end poly(A)s sufficient for rescuing Senecavirus A from its cDNA clone, but inadequate to meet requirement of viral replication

**DOI:** 10.1016/j.virusres.2023.199076

**Published:** 2023-03-01

**Authors:** Di Zhao, Yan Li, Ziwei Li, Lijie Zhu, Yuxuan Sang, Hui Zhang, Feng Zhang, Bo Ni, Fuxiao Liu

**Affiliations:** a College of Veterinary Medicine, Qingdao Agricultural University, Qingdao, 266109, China; b College of Veterinary Medicine, Inner Mongolia Agricultural University, Huhhot, 010018, China; c Qingdao Center for Animal Disease Control & Prevention, Qingdao, 266199, China; d Surveillance Laboratory of Livestock Diseases, China Animal Health and Epidemiology Center, Qingdao, 266032, China

**Keywords:** Senecavirus A, 3′ end, poly(A) tail, cDNA clone, Virus rescue, eGFP

## Abstract

Senecavirus A (SVA) belongs to the genus *Senecavirus* in the family *Picornaviridae*. Its genome is a positive-sense, single-strand RNA that has 5′ and 3′ untranslated regions. There is a poly(A) tail at the 3′ end of viral genome. Although the number of poly(A)s is variable, the length of poly(A) tail generally has the minimum nucleotide limit for picornaviral replication. To identify a range limit of poly(A)s for SVA recovery, five SVA cDNA clones, separately containing 25, 20, 15, 10 and 5 poly(A)s, were constructed for rescuing viruses. Replication-competent SVAs could be rescued from the first three cDNA clones, implying the range limit of poly(A)s was (A)_15_ to (A)_10_. To recognize the precise limit, four extra cDNA clones, separately containing 14, 13, 12 and 11 poly(A)s, were constructed to rescue SVAs further. The replication-competent SVA was rescued only from the poly(A)_14_-containing plasmid, indicating that the precise limit was poly(A)_14_ at the 3′ end of cDNA clone for SVA recovery. The rescued SVA was serially passaged in cells. The passage-5 and -10 progenies were independently subjected to the analysis of 3′-rapid amplification of cDNA ends. Both progenies showed their own poly(A) tails far more than 14 (A)s, implying extra (A)s added to the poly(A)_14_ sequence during viral passaging. It can be concluded that fourteen (A)s are sufficient for rescuing a replication-competent SVA from its cDNA clone, but inadequate for maintaining viral propagation in cells.

## Highlights

• Fourteen 3′-end poly(A)s are sufficient for rescuing Senecavirus A (SVA).
• Fourteen 3′-end poly(A)s are inadequate to meet requirement of SVA replication.
• Length of poly(A) tail is unstable during SVA replication.

## 1 Introduction

Seneca Valley virus, currently renamed Senecavirus A (SVA), is an emerging virus, causing vesicular disease and epidemic transient neonatal losses in pigs (Liu et al., 2020b). SVA-induced vesicular lesion is indistinguishable from clinical signs caused by other vesicular diseases, such as foot-and-mouth disease (Sturos et al., 2022). SVA infection has been reported in several countries, like Canada (Pasma et al., 2008), the USA (Singh et al., 2012), Brazil (Vannucci et al., 2015), China (Wu et al., 2017) and Thailand (Saeng-Chuto et al., 2018). SVA infection is one of the major concerns owing to its negative impact on affected pig farms. Due to lack of efficient treatment and vaccination, it has been difficult to effectively control such an emerging disease worldwide.

SVA is classified taxonomically to the genus *Senecavirus* in the family *Picornaviridae*. Its genome is a positive-sense, single-strand RNA, approximately 7300 nucleotides in length, which contains 5′ and 3′ untranslated regions (UTRs), and a single open reading frame (ORF) of polyprotein precursor. The 5′ and 3′ UTRs harbor an internal ribosome entry site (IRES) (Willcocks et al., 2011) and a putative pseudoknot (or kissing-loop) structure (Liu et al., 2022), respectively. Referring to those of other picornaviruses (Sun et al., 2016), the polyprotein precursor of SVA is encoded and further cleaved stepwisely into twelve polypeptides: L, VP4, VP2, VP3, VP1, 2A, 2B, 2C, 3A, 3B, 3C^pro^ and 3D^pol^. The picornaviral genome is typically an mRNA that has a polyadenylic acid [poly(A)] tail at the 3′ end, but no 5′-capped structure. As a substitute, the VPg (or 3B) is covalently linked to the 5′ end for initiating viral replication through functioning as a protein primer for RNA synthesis (Gavryushina et al., 2011; Paul and Wimmer, 2015).

The poly(A) tail was reported to exist at the 3′ end of picornavirus (poliovirus) as early as 1972 (Yogo and Wimmer, 1972). The poly(A) structure plays a vital role in the replication of picornaviral antigenome. It has been demonstrated that poly(A)s at the 3′ end of polioviral positive-strand RNA are transcribed into a VPg-linked poly(U) product at the 5′ end of negative-strand RNA during RNA replication, and then VPg-linked poly(U) sequences at the 5′ ends of negative-strand RNA templates are transcribed into poly(A) sequences at the 3′ ends of positive-strand RNAs. Besides as a template for synthesizing the VPg-linked poly(U)s, the polioviral poly(A) tail is additionally proven to be necessary for generating a circularized ribonucleoprotein complex, which formed around the 5′-end cloverleaf structure interacts with the poly(A) binding protein (PABP) bound to the 3′ poly(A) tail, therefore linking the ends of the viral RNA, called genome circularization *via* a protein-protein bridge (Herold and Andino, 2001).

The poly(A) tails are essential for picornaviral viability. Their lengths, albeit variable (Ahlquist and Kaesberg, 1979), affect the magnitudes of both viral mRNA translation and RNA replication (Silvestri et al., 2006). Reduction of the poly(A) length noticeably decreases the specific infectivity of poliovirus RNA (Spector and Baltimore, 1974), as evidenced by the fact that the RNA with a poly(A)_12_ tail is only 10% as infectious as virion RNAs (Sarnow, 1989). In addition, negative-strand RNA synthesis by the transcript RNA with a poly(A)_12_ tail is significantly reduced, compared with that by another transcript RNA with a poly(A)_83_ tail (Barton et al., 1996). In contrast, increasing the length of the polioviral poly(A) tail from (A)_12_ to (A)_13_ results in approximately a 10-fold increase in negative-strand synthesis (Silvestri et al., 2006).

The role of polioviral poly(A) tail has been widely explored over four decades. As an emerging picornavirus, SVA has not been deeply studied as yet. It remains still unclear that the least number of poly(A)s is required for rescuing a replication-competent SVA from its cDNA clone. It is also unclear whether a poly(A) tail with the minimum (A)s is genetically stable during SVA passaging in cells. This prompted us to conduct this study using reverse genetics for clarifying these two basic issues.

## 2 Materials and methods

### 2.1 Cell and plasmid

The BSR-T7/5 cell line has been extensively used for reverse genetic manipulation. It was cultured at 37°C with 5% CO_2_ in Dulbecco's modified Eagle's medium (DMEM), supplemented with 10% fetal bovine serum (VivaCell, Shanghai, China), penicillin (100 U/mL), streptomycin (100 µg/mL), amphotericin B (0.25 µg/mL) and G418 (500 µg/mL). The plasmid, containing one SVA's full-length cDNA clone tagged with an sequence of enhanced green fluorescent protein (eGFP), was constructed previously (Liu et al., 2020a). This cDNA clone was a wild-type one that had a total of thirty 3′-end poly(A)s, and was regulated by the T7 promoter in the plasmid (named p30A).

### 2.2 Construction of multi(A)-deleting SVA cDNA clones

Multiple (A)s were gradually deleted from the poly(A) sequence in the wild-type cDNA clone for constructing multi(A)-deleting plasmids, named pXA [X = No. of poly(A)s retained in cDNA clone]. Briefly, multi(A)-deleting DNA sequences, containing the *Sbf* I and the *Pme* I sites, were chemically synthesized, and independently subcloned into the pUC57 plasmid. These recombinant pUC57 plasmids were used as PCR templates to amplify multi(A)-deleting fragments, using the forward (cgagtcacgagtaCCTGCAGGca) and the reverse (ctgatcagcggGTTTAAACgggc) primers. The *Sbf* I and *Pme* I sites were shown by uppercase sequences in the forward and reverse primers, respectively. The PCR reaction contained 2 × PrimeSTAR Max Premix (Takara, Dalian, China), and underwent 35 cycles at 98°C (10 s), 58°C (5 s) and 72°C (5 s). The PCR products were purified from an agarose gel for subcloning into the *Sbf* I/*Pme* I-digested p30A to construct separately multi(A)-deleting mutants, using the In-Fusion® Kit (Takara, Dalian, China) according to the manufacturer's instruction. All mutants were confirmed by Sanger sequencing, and then purified using the SPARKeasy Superpure Mini Plasmid Kit (Shandong Sparkjade Biotechnology Co., Ltd.).

### 2.3 Rescue of recombinant SVAs (rSVAs)

BSR-T7/5 cells were seeded into a 24-well plate, and then cultured at 37°C with 5% CO_2_. Cell monolayers at 70% confluency were independently transfected with multi(A)-deleting plasmids (1000 ng/well) using Lipofectamine 2000 (Thermo Fisher, Waltham, MA, USA) according to the manufacturer's instruction. The plasmid-transfected cell monolayers were cultured at 37°C with 5% CO_2_, and observed using a fluorescence microscope at 72 h post-transfection (hpt). Culture supernatants were harvested after one freeze-thaw cycle for serial blind passages in BSR-T7/5 cells. Supernatant-inoculated cell monolayers were observed under the fluorescence microscope at each viral passage. Green fluorescence served as a marker to indicate whether a given replication-competent SVA could be rescued from its own cDNA clone. If so, it would be named rSVA-XA [X = No. of poly(A)s retained in cDNA clone].

### 2.4 RT-PCR detection of rSVAs

The culture supernatants were independently harvested at passage-5 (P5) for extracting total RNAs using the Viral RNA/DNA Extraction Kit (Takara, Dalian, China). The extracted products were used as templates for one-step RT-PCR analysis and additionally for PCR detection, using the PrimeScript™ High Fidelity One Step RT-PCR Kit (Takara, Dalian, China) and the Phanta® Flash Master Mix (Vazyme, Nanjing, China), respectively. In brief, the RT-PCR underwent 45°C for 10 min, 94°C for 2 min and then 30 cycles at 98°C (10 s), 55°C (15 s) and 68°C (10 s) using the forward (5′-AGGCACAGAGGAGCAACATCCAA-3′) and reverse (5′-ATCGTTCACCGATCTAGGGTATT-3′) primers (Liu et al., 2020a). The PCR underwent 30 cycles at 98°C (10 s), 55°C (5 s) and 72°C (10 s) using the same pair of primers. RT-PCR and PCR products were simultaneously detected by agarose gel electrophoresis.

### 2.5 3′-rapid amplification of cDNA ends (3′-RACE)

The rSVA, rescued from a cDNA clone with the minimum number of poly(A)s, was serially passaged *in vitro*. The P5 and P10 progenies were analyzed by 3′-RACE reaction using the HiScript-TS 5′/3′ RACE Kit (Vazyme, Nanjing, China) according to the manufacturer's instruction. Two 3′-RACE products were independently subcloned into linear plasmids using the TA/Blunt-Zero Cloning Kit (Vazyme, Nanjing, China) for bacterial transformation. Four single colonies were picked from each agar plate for Sanger sequencing.

## 3 Results and discussion

**Fig. 1:**
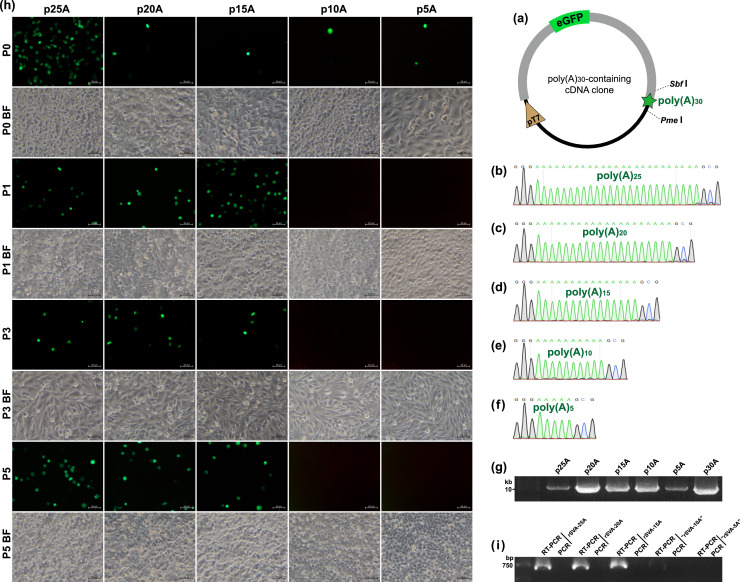
Construction of five SVA cDNA clones with curtailed poly(A) tails for rescuing replication-competent viruses. Schematic representation of plasmid containing the wild-type SVA cDNA clone tagged with eGFP sequence. *Sbf* I/*Pme* I-targeted sites are indicated with two dotted lines. The proportion of elements does not exactly match them in the plasmid. Sanger sequencing chromatograms of five multi(A)-deleting cDNA clones, *i.e.*, p25A (b), p20A (c), p15A (d), p10A (e) and p5A (f). Agarose gel electrophoresis of five plasmids after purification (g). Profile of eGFP expression in BSR-T7/5 cells at P0, P1, P3 and P5 (h). Cell monolayers are independently transfected with five genetically modified plasmids, and then subjected to one freeze-and-thaw cycle at 72 hpt (P0) to harvest culture supernatants for five serial passages. BF: bright field. RT-PCR detection of rSVA-25A, -20A, -15A, -“10A” and -“5A” at P5 (i). PCR analysis is simultaneously performed to demonstrate no interference of plasmid residues.

It was generally considered that SVA was originally identified in 2002 as a contaminant in cell culture (Hales et al., 2008). This virus later is found to be able of inducing vesicular disease in pigs. Now, SVA is still regarded as an emerging virus worldwide. To facilitate our studies on its molecular mechanisms, the system of SVA reverse genetics had been established previously (Liu et al., 2020a), composed of the p30A that contained an eGFP-tagged cDNA clone (Fig. 1a), and the BSR-T7/5 cell line in which the T7 RNA polymerase was constitutively expressed. The full-length sequence of cDNA clone, derived from that of the isolate (GenBank: KX751945.1), bore a poly(A)_30_ tail at its 3′ end. We speculated the poly(A)_30_ tail necessary for virus recovery from the full-length cDNA clone. If consecutive multiple (A)s were deleted from the plasmid, the limit of tolerance to nucleotide deletion would be accurately identified for virus rescue. It is commonly time- and labor-consuming that a single (A) is deleted one by one from the plasmid. Therefore, five multi(A)-deleting plasmids, namely p25A (Fig. 1b), p20A (Fig. 1c), p15A (Fig. 1d), p10A (Fig. 1e) and p5A (Fig. 1f), were firstly constructed for recognizing a range limit of tolerance to nucleotide deletion for rSVA rescue.

These five plasmids were extracted for Sanger sequencing (Fig. 1b to f) and agarose gel electrophoresis (Fig. 1g), and then independently transfected into BSR-T7/5 cells in an attempt to rescue rSVAs. Green fluorescence was visible on all plasmid-transfected cell monolayers at 72 hpt (Fig. 1h, Panel P0), whereas only three groups (p25A, p20A and p15A) always exhibited the fluorescence-emitted phenotype during serial blind passages (Fig. 1h, Panel P1, P3 and P5). Green fluorescence was an ideal marker here that indicated whether a given rSVA had been recovered or not. Thus, it could be concluded that neither rSVA-10A nor -5A was rescued, due to fluorescence-emitted phenotype unobservable with blind passaging. To confirm this conclusion, all samples were collected after five serial passages *in vitro* for RT-PCR detection. The result showed three bands with the expected size (approximately 700 bp) on a gel (Fig. 1i, RT-PCR lanes). The test of PCR control indicated no cDNA plasmid residue affecting RT-PCR detection (Fig. 1i, PCR lanes). The RT-PCR analysis confirmed the rSVA-25A, -20A and -15A rescued from their individual cDNA clones, implying the range limit of tolerance to nucleotide deletion was 11 to 14 (A)s for virus rescue.

**Fig. 2:**
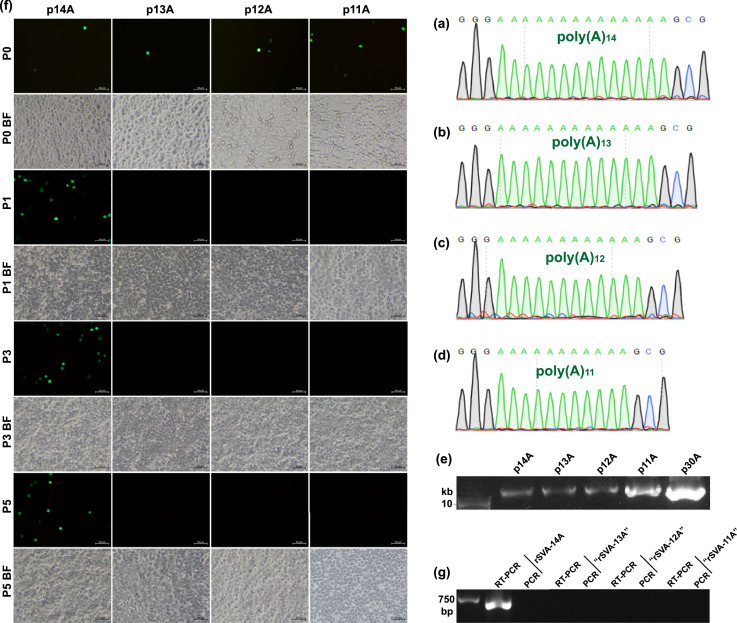
Construction of four SVA cDNA clones with curtailed poly(A) tails for rescuing replication-competent viruses. Sanger sequencing chromatograms of four multi(A)-deleting cDNA clones, *i.e.*, p14A (a), p13A (b), p12A (c) and p11A (d). Agarose gel electrophoresis of four plasmids after purification (e). Profile of eGFP expression in BSR-T7/5 cells at P0, P1, P3 and P5 (f). Cell monolayers are independently transfected with p14A, p13A, p12A and p11A for further culturing at 37°C with 5% CO_2_. Plasmid-transfected cell cultures undergo one freeze-and-thaw cycle at 72 hpt (P0) to harvest supernatants for five serial passages. BF: bright field. RT-PCR detection of rSVA-14A, -“13A”, -“12A” and -“11A” at P5 (g). PCR analysis is simultaneously performed to demonstrate no interference of plasmid residues.

In order to recognize a precise limit here, four extra multi(A)-deleting cDNA clones, p14A (Fig. 2a), p13A (Fig. 2b), p12A (Fig. 2c) and p11A (Fig. 2d), were constructed, and subsequently underwent Sanger sequencing (Fig. 2a to d) and agarose gel electrophoresis (Fig. 2e). Four plasmids were separately transfected into BSR-T7/5 cells to rescue rSVAs. Only the group p14A always showed its own fluorescence-emitted phenotype with serial passaging (Fig. 2f, Panel P1, P3 and P5). The RT-PCR detection (Fig. 2g) confirmed that only the rSVA-14A was successfully rescued, suggesting at least fourteen 3′-end poly(A)s required for rescuing the replication-competent SVA from its cDNA clone.

**Fig. 3:**
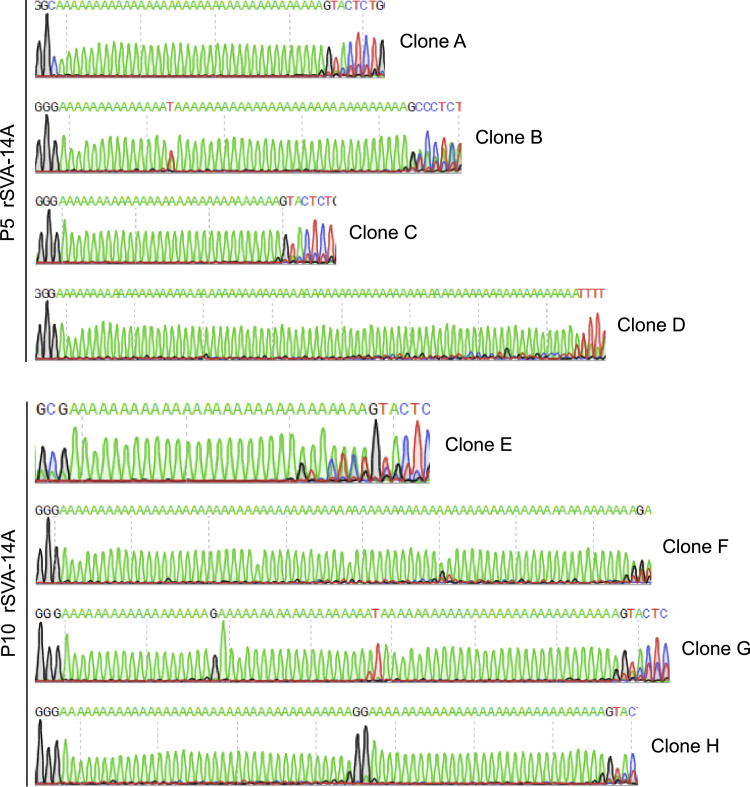
Sanger sequencing chromatograms of 3′-RACE products amplified from rSVA-14A at P5 (Clone A to D) and P10 (Clone E to H).

The rSVA-14A underwent ten serial passages *in vitro*, and always expressed the eGFP in cells during passaging (Supplementary 1). In order to determine the stability of genetically modified poly(A) tail, the number of poly(A)s was experimentally measured at P5 and P10 using the 3′-RACE. Unfortunately, the exact number failed to be measured, as evidenced by different 3′-RACE clones with differential poly(A) sequences even at the same passage (Fig. 3). Interestingly, all of the eight 3′-RACE clones (Fig. 3, Clone A to H) showed their own poly(A) tails far more than 14 (A)s, suggesting that extra (A)s were added to the poly(A)_14_ sequence with viral passaging. In other words, fourteen (A)s were sufficient for the recovery of an rSVA from its cDNA clone, but inadequate to meet the requirement of viral replication in cells.

The accurate relationship, between the 3′-end poly(A) tail and the infectivity of poliovirus RNA, has been extensively studied, as described in subheading Introduction. Silvestri *et al* (2006) demonstrated that increasing the poly(A) length from (A)_12_ to (A)_20_ led to an dramatic increase in negative-strand RNA synthesis independent of RNA stability or translation. They additionally observed a slight rise in PABP-mediated binding, when the length of the poly(A) tail increased from (A)_12_ to (A)_20_. Therefore, besides its interaction with PABPs, the polioviral poly(A) tail played a direct role in the initiation of negative-strand RNA synthesis (Silvestri et al., 2006). Duck hepatitis A virus type 1 (picornavirus) was more recently demonstrated that at least (A)_20_ were required for the optimal genome replication. The virus replication was severely impaired when the poly(A) tail was curtailed to (A)_10_ (Chen et al., 2018). Picornaviral IRES-dependent translation had been shown to be stimulated by poly(A) sequences in cell-free extracts (Bergamini et al., 2000; Michel et al., 2001; Svitkin et al., 2001). In these cases, a model of protein-protein bridge was proposed to mediate the poly(A)-related stimulation. Besides the poly(A) tail, the 3′ UTR was also involved in stimulating the IRES-driven translation (López de Quinto et al., 2002).

As mentioned above, the picornaviral poly(A) tails play two major roles, namely initiating anti-genome replication and mediating protein translation, in viral propagation. In the present study, at least (A)_14_ at 3′ end were required for rescuing the replication-competent SVA from a cDNA clone. Like those of other picornaviruses, the poly(A) tail of SVA also acts as a *cis*-acting RNA element during viral propagation. Here, we speculate that the poly(A) tail, if less than fourteen (A)s, would not interact with one or more *trans*-acting factors, like the viral 3D^pol^ and the cellular PABP, leading to the failure of SVA recovery from a certain cDNA clone. Even though successfully rescued, the rSVA-14A unexpectedly showed a mutated genotype with a prolonged poly(A) tail. It remains to be elucidated how extra (A)s are added to the original poly(A)_14_ sequence during viral replication.

## CRediT authorship contribution statement

**Di Zhao:** Methodology. **Yan Li:** Methodology. **Ziwei Li:** Methodology, Software. **Lijie Zhu:** Methodology, Software. **Yuxuan Sang:** Methodology, Software. **Hui Zhang:** Formal analysis, Writing – review & editing. **Feng Zhang:** Formal analysis, Writing – review & editing. **Bo Ni:** Writing – review & editing. **Fuxiao Liu:** Writing – review & editing.

## Competing interests

The authors declare that they have no competing interests.

## Data Availability

Data will be made available on request.

## Appendix Supplementary materials

Image, application 1

## References

[bib0001] Ahlquist P., Kaesberg P. (1979). Determination of the length distribution of poly(A) at the 3′ terminus of the virion RNAs of EMC virus, poliovirus, rhinovirus, RAV-61 and CPMV and of mouse globin mRNA. Nucl. Acids Res.

[bib0002] Barton D.J., Morasco B.J., Flanegan J.B. (1996). Assays for poliovirus polymerase, 3D(Pol), and authentic RNA replication in HeLa S10 extracts. Methods Enzymol.

[bib0003] Bergamini G., Preiss T., Hentze M.W. (2000). Picornavirus IRESes and the poly(A) tail jointly promote cap-independent translation in a mammalian cell-free system. RNA.

[bib0004] Chen J.H., Zhang R.H., Lin S.L., Li P.F., Lan J.J., Song S.S., Gao J.M., Wang Y., Xie Z.J., Li F.C., Jiang S.J. (2018). The functional role of the 3′ untranslated region and Poly(A) tail of duck hepatitis A virus Type 1 in viral replication and regulation of IRES-Mediated translation. Front. Microbiol..

[bib0005] Gavryushina E.S., Bryantseva S.A., Nadezhdina E.S., Zatsepin T.S., Toropygin I.Y., Pickl-Herk A., Blaas D., Drygin Y.F. (2011). Immunolocalization of Picornavirus RNA in infected cells with antibodies to Tyr-pUp, the covalent linkage unit between VPg and RNA. J. Virol. Methods.

[bib0006] Hales L.M., Knowles N.J., Reddy P.S., Xu L., Hay C., Hallenbeck P.L. (2008). Complete genome sequence analysis of Seneca Valley virus-001, a novel oncolytic picornavirus. J. Gen. Virol..

[bib0007] Herold J., Andino R. (2001). Poliovirus RNA replication requires genome circularization through a protein-protein bridge. Mol. Cell.

[bib0008] López de Quinto S., Sáiz M., de la Morena D., Sobrino F., Martínez-Salas E. (2002). IRES-driven translation is stimulated separately by the FMDV 3′-NCR and poly(A) sequences. Nucleic Acids Res.

[bib0009] Liu F., Huang Y., Wang Q., Shan H. (2020). Construction of eGFP-tagged senecavirus a for facilitating virus neutralization test and antiviral assay. Viruses.

[bib0010] Liu F., Wang Q., Huang Y., Wang N., Shan H. (2020). A 5-year review of senecavirus A in China since its emergence in 2015. Front. Vet. Sci..

[bib0011] Liu F., Zhao D., Wang N., Li Z., Dong Y., Liu S., Zhang F., Cui J., Meng H., Ni B., Wei R., Shan H. (2022). Tolerance of senecavirus A to mutations in its kissing-loop or pseudoknot structure computationally predicted in 3′ untranslated region. Front Microbiol.

[bib0012] Michel Y.M., Borman A.M., Paulous S., Kean K.M. (2001). Eukaryotic initiation factor 4G-poly(A) binding protein interaction is required for poly(A) tail-mediated stimulation of picornavirus internal ribosome entry segment-driven translation but not for X-mediated stimulation of hepatitis C virus translation. Mol. Cell. Biol..

[bib0013] Pasma T., Davidson S., Shaw S.L. (2008). Idiopathic vesicular disease in swine in Manitoba. Can. Vet. J..

[bib0014] Paul A.V., Wimmer E. (2015). Initiation of protein-primed picornavirus RNA synthesis. Virus Res.

[bib0015] Saeng-Chuto K., Rodtian P., Temeeyasen G., Wegner M., Nilubol D. (2018). The first detection of Senecavirus A in pigs in Thailand, 2016. Transbound Emerg. Dis..

[bib0016] Sarnow P. (1989). Role of 3′-end sequences in infectivity of poliovirus transcripts made in vitro. J. Virol..

[bib0017] Silvestri L.S., Parilla J.M., Morasco B.J., Ogram S.A., Flanegan J.B. (2006). Relationship between poliovirus negative-strand RNA synthesis and the length of the 3′ poly(A) tail. Virology.

[bib0018] Singh K., Corner S., Clark S., Scherba G., Fredrickson R. (2012). Seneca valley virus and vesicular lesions in a pig with idiopathic vesicular disease. J. Vet. Sci. Technol..

[bib0019] Spector D.H., Baltimore D. (1974). Requirement of 3′-terminal poly(adenylic acid) for the infectivity of poliovirus RNA. Proc. Natl. Acad. Sci. U. S. A..

[bib0020] Sturos M.J., Murray D., Johnson L., Preis G., Corzo C.A., Rossow S., Vannucci F.A. (2022). Persistence and shedding of senecavirus A in naturally infected boars. J. Vet. Diagn. Invest..

[bib0021] Sun D., Chen S., Cheng A., Wang M. (2016). Roles of the picornaviral 3C proteinase in the viral life cycle and host cells. Viruses.

[bib0022] Svitkin Y.V., Imataka H., Khaleghpour K., Kahvejian A., Liebig H.D., Sonenberg N. (2001). Poly(A)-binding protein interaction with elF4G stimulates picornavirus IRES-dependent translation. RNA.

[bib0023] Vannucci F.A., Linhares D.C., Barcellos D.E., Lam H.C., Collins J., Marthaler D. (2015). Identification and complete genome of seneca valley virus in vesicular fluid and sera of pigs affected with idiopathic vesicular disease, Brazil. Transbound Emerg. Dis..

[bib0024] Willcocks M.M., Locker N., Gomwalk Z., Royall E., Bakhshesh M., Belsham G.J., Idamakanti N., Burroughs K.D., Reddy P.S., Hallenbeck P.L., Roberts L.O. (2011). Structural features of the Seneca Valley virus internal ribosome entry site (IRES) element: a picornavirus with a pestivirus-like IRES. J. Virol..

[bib0025] Wu Q., Zhao X., Bai Y., Sun B., Xie Q., Ma J. (2017). The first identification and complete genome of Senecavirus A affecting pig with idiopathic vesicular disease in China. Transbound Emerg Dis.

[bib0026] Yogo Y., Wimmer E. (1972). Polyadenylic acid at the 3′-terminus of poliovirus RNA. Proc. Natl. Acad. Sci. U. S. A..

