# Supplementary material for: Only fourteen 3′-end poly(A)s sufficient for rescuing Senecavirus A from its cDNA clone, but inadequate to meet requirement of viral replication
Source: Virus Res. 2023 Mar 1;328:199076. doi: 10.1016/j.virusres.2023.199076 (PMC10194250; doi:10.1016/j.virusres.2023.199076)
Supplement: Supplementary file 1 [file mmc1.docx]

**Supplementary 1** Profile of rSVA-14A-mediated eGFP expression in BSR-T7/5 cells at P6, P7, P8, P9 and P10.
